# Assessment of Nutrition Status and Its Effect on Outcomes in Patients With Limb Injuries Using the Subjective Global Assessment as a Screening Tool

**DOI:** 10.7759/cureus.44953

**Published:** 2023-09-09

**Authors:** Narendra Singh Kushwaha, Divya Bhanu Rana, Arpit Singh, Suraj Saxena, Shubham Srivastava, Vineet Sharma

**Affiliations:** 1 Department of Orthopaedic Surgery, King George’s Medical University, Lucknow, IND

**Keywords:** subjective global assessment, bone healing, rust scoring system, limb injuries, nutritional status

## Abstract

Background

Malnutrition in hospitalized patients is a significant problem. This study aimed to assess the utility of the Subjective Global Assessment (SGA) in predicting the association between serum biomarkers and malnutrition in patients with limb injuries as well as the impact of malnutrition on clinical and radiological bone healing.

Methodology

This prospective study included 93 patients with limb injuries. Basic demographic details, serum biomarker levels, nutritional status assessed using the SGA, and the correlation of the Radiological Union Shaft Tibia (RUST) score with nutrition status were assessed along with the secondary outcomes.

Results

According to the SGA, patients were classified into Group A (well-nourished), Group B (moderately malnourished), and Group C (severely malnourished). Serum biomarkers (albumin, hemoglobin, platelets, and total leucocyte count) were significantly higher in Group A than in Group B + C (p < 0.0001). The nutritional status of patients from admission up to six months in Group A was significantly higher (p < 0.0001) compared to Group B + C. The radiological healing according to the RUST score had a negative correlation with C-reactive protein and a positive correlation with various parameters at six months.

Conclusions

The serum biomarker levels and the clinical and radiological bone healing, as measured by the RUST scoring system, showed a positive correlation with the nutritional status of the patients. Malnutrition significantly increases the chance of developing complications such as wound infection, decubitus, and infected implants.

## Introduction

Malnutrition is defined as an imbalance in nutrition due to inadequate nutrient intake or inadequate utilization or absorption of ingested nutrients, resulting in altered body composition (decreased fat-free mass and decreased body cell mass) and diminished body function (such as muscular performance, organ function, body composition, and functional capacity) [1].

Malnutrition is frequently underdiagnosed in surgical patients and is considered to be a factor in poorer outcomes during hospitalization or after fracture surgery. Malnutrition is estimated to affect 50% of hospitalized adult patients, making it one of the most prevalent comorbidities in this population [2-5]. Special attention has been paid to malnutrition in orthopedic patients [6,7]. There is a significant correlation between subnormal nutrition indices and the onset of complications [6].

Malnutrition plays an essential role in the development of complications, including infection and delayed wound and fracture healing, in orthopedic surgical patients [7]. Malnutrition in orthopedic patients, an overlooked and understudied condition, has suboptimal outcomes.

Unfortunately, malnourished hospitalized trauma patients frequently struggle to maintain a healthy nutritional status. The reported prevalence of malnutrition among hospitalized patients ranges from 10% to 50%, depending on the study population [8-12]. In the general surgery literature, malnutrition has been linked to increased rates of complications and mortality after abdominal surgeries [9,10]. These findings are consistent with the literature on orthopedic surgery.

Therefore, adequate nutrition is essential for musculoskeletal healing. Hence, it is reasonable to assume that poor nutrition may also contribute to higher rates of delayed union and nonunion [11-14]. Several studies on animal models of long bone fractures have demonstrated that nutritional optimization improves fracture healing [15,16], but the literature on delayed fracture healing and nutritional status in humans is limited. In addition, the available limited literature on nutritional status and outcomes after orthopedic trauma is highly inconsistent. Multiple sources have noted several patient traits linked to a higher risk of infection following surgical intervention. These traits include diabetes, systemic steroid usage, remote site infections, malnutrition, chronic renal failure, a history of open surgery, morbid obesity, smoking, and drinking [13].

## Materials and methods

This prospective study was conducted at the Department of Orthopaedics, King George’s Medical University, Lucknow, India, for a period of one year. Study approval was obtained from the Institutional Ethics Committee of King George’s Medical University (approval number: IX-PGTSC-IIA/P8). The sample size was calculated based on a previous study that reported a 40% prevalence of malnutrition in severely injured patients [17]. Patients with limb injuries who were admitted to the Department of Orthopaedic Surgery, King George’s Medical University, from 2020 onwards were enrolled in the study. Patients who had undergone any other surgery likely to affect the physical status of the patient and those with a history of treatment for any other condition likely to affect their physical or mental well-being (e.g., depression, schizophrenia, mania, chronic painful conditions, acute neuralgic pain) were excluded from the study. Written consent was taken from patients willing to participate in the study. Patients were physically assessed at the time of admission, and their age, sex, diagnosis, serum biomarkers (albumin, C-reactive protein (CRP)), and complete blood count were noted. Nutrition-related history according to the Subjective Global Assessment (SGA) protocol was obtained from all patients. Data from the SGA history were summarized into one of the following three groups: Group A (well-nourished), Group B (moderately (or suspected of being) malnourished), and Group C (severely malnourished). Follow-up was done at one week, at discharge, and at one month, three months, and six months post-discharge to record the following parameters: SGA, serum biomarkers, and bone healing. Radiological signs of healing were evaluated using the Radiological Union Shaft Tibia (RUST) score. Secondary outcomes such as wound infection, sepsis, implant infection, decubitus, need for intensive care unit, death, and any other complications were evaluated among the groups. Data were collected using a structured proforma, entered in an MS Excel sheet, and analyzed using SPSS version 26.0 (IBM Corp., Armonk, NY, USA). Qualitative data were expressed as proportions.

## Results

A total of 93 patients of both sexes and above 18 years of age with limb injuries were enrolled according to the exclusion and inclusion criteria. All enrolled patients were divided into two groups per the SGA (Group A = 48 and Group B + C = 45). On analyzing the age distribution, the mean age of Group A (40.42 ± 17.22 years) was higher than the combined age of Group B + C (39.09 ± 18.50), with a statistically insignificant difference among them (p = 0.7204). Regarding the gender of patients, male dominance was observed in both groups with no significant difference (p = 0.9362). Serum biomarker levels among the enrolled patients showed higher levels of albumin, CRP, hemoglobin, platelet count, and total leucocyte count (TLC) with highly significant differences among the groups (Table 1).

**Table 1:** Serum biomarker levels in enrolled patients. Significant p-value <0.001.

Serum biomarkers	Group A	Group B + C	P-value
	Mean ± SD	Mean ± SD	
Albumin	4.09 ± 0.71	0.92 ± 0.34	<0.0001*
C-reactive protein	2.57 ± 0.43	15.51 ± 10.79	<0.0001*
Hemoglobin	13.60 ± 0.95	9.40 ± 1.03	<0.0001*
Platelets count	194,502.13 ± 56,011.71	127,998.67 ± 15,108.34	<0.0001*
Total leukocyte count	7,130.75 ± 1,969.82	3,868.27 ± 1,129.33	<0.0001*

The nutritional status of patients per the SGA among the groups at different follow-ups was observed to be highly significant from admission to the sixth-month follow-up. The mean score at admission was higher in Group A (6.25 ± 0.43) than in Group B + C (2.93 ± 1.06), with a statistically significant difference among them (p < 0.0001). In Group A, most patients were well-nourished (36, 75%); however, in Group B + C, most patients were mild-to-moderate malnourished (21, 46.67%), with a statistically significant difference among them (p < 0.0001) (Table 2).

**Table 2:** Comparison of nutritional status at different follow-ups. Significant p-value <0.001.

Nutritional status	Group A (mean ± SD)	Group B+C (mean ± SD)	P-value
At admission	6.25 ± 0.43	2.93 ± 1.06	<0.0001*
At discharge	6.25 ± 0.43	2.93 ± 1.06	<0.0001*
At 1 week	6.25 ± 0.43	2.93 ± 1.06	<0.0001*
1^st^ month	6.31 ± 0.46	3.02 ± 1.16	<0.0001*
3^rd^ month	6.44 ± 0.50	4.20 ± 1.56	<0.0001*
6^th^ month	6.65 ± 0.48	4.93 ± 1.34	<0.0001*

**Figure 1:**
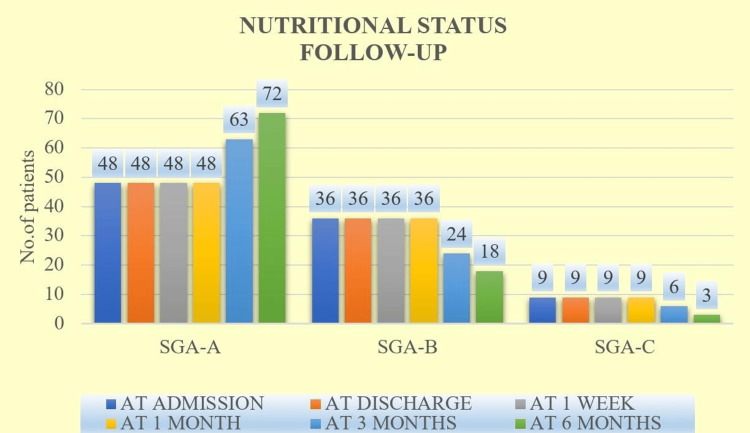
Comparative nutritional status for total enrolled patients.

Comparative nutritional status at follow-up is shown in Figure 1.

Secondary outcomes were observed to be maximum in Group B + C compared to Group A. Further, in Group A, the maximum number of patients reported having wound infection (16, 33.33%). In Group B + C, the maximum number of patients reported having wound infection (45, 100%), followed by decubitus (38, 79.17%). Furthermore, statistically significant differences were observed in both groups for all observed secondary outcomes (Table 3).

**Table 3:** Outcomes observed in the enrolled groups. Significant p-value <0.001.

Secondary outcome	Group A		Group B + C		P-value
	N	%	N	%	
Wound infection	16	33.33%	45	100.00%	<0.0001*
Decubitus	9	20.00%	38	79.17%	<0.0001*
Infected implant	6	12.50%	18	40.00%	0.0025*
Ileus	3	6.25%	18	40.00%	0.0001*
Any other complications	3	6.25%	18	40.00%	0.0001*

Correlation analysis of the RUST score at six months with various parameters

**Figure 2:**
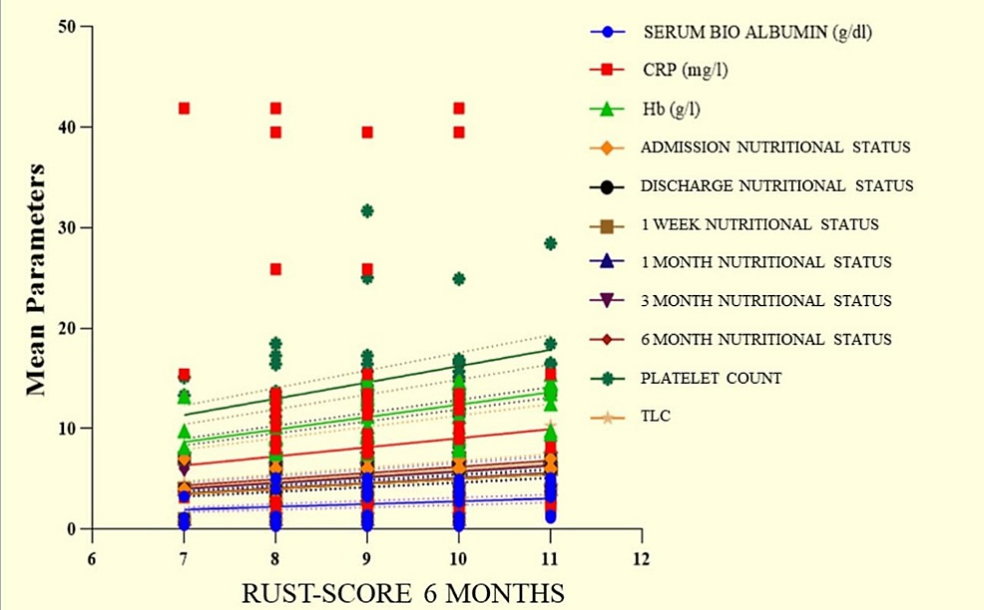
The correlation analysis of the RUST score at six months with various parameters. RUST: Radiological Union Shaft Tibia

On analyzing the RUST score with various parameters at six months, we observed a positive correlation with a significant association between the RUST score versus serum bio albumin (r = 0.3072; p = 0.0027), hemoglobin (r = 0.3411; p = 0.0135), platelet count (r = 0.3389; p = 0.0471), TLC (r = 0.2579; p = 0.0125), on-admission nutritional status (r = 0.3473; p = 0.0006), on-discharge nutritional status (r = 0.3473; p = 0.0006), one-week nutritional status (r = 0.3473; p = 0.0006), one-month nutritional status (r = 0.3666; p = 0.0003), three-month nutritional status (r = 0.4018; p < 0.0001), and six-month nutritional status (r = 0.442; p < 0.0001). A negative correlation with a significant association was observed between the RUST score versus CRP (r = -0.2911; p = 0.0046). The correlation analysis is shown in Figure 2.

## Discussion

The present observational, hospital-based, prospective study was performed among all patients attending the outpatient or inpatient department of the Department of Orthopaedic Surgery, King George’s Medical University, Lucknow willing to participate in the study. After obtaining ethical clearance and consent forms, 93 cases with limb injuries of both sexes and above 18 years were enrolled per the exclusion and inclusion criteria. All enrolled patients were divided into two groups per the SGA (Group A = 48 and Group B + C = 45).

A common comorbidity in this population was malnutrition, which is estimated to affect 50% of adult hospital patients. Correia et al. [2] reported that in surgical patients, malnutrition is usually underdiagnosed and is thought to contribute to worse outcomes during hospitalization or following fracture repair. Malnutrition in orthopedic patients is given particular consideration.

On analyzing the age distribution of patients, we observed that the mean age of Group A (40.42 ± 17.22) was more than the combined age of Group B + C (39.09 ± 18.50). Similarly, in Group B, the maximum number of patients in Group A were in the age group of 31-42 years (18 (37.50%)). However, the maximum number of patients in Group B were in the age group of 19-30 years (19 (42.22%)), with a statistically insignificant difference.

The gender distribution in our patients had a male dominance in both Group A and Group B + C with male-to-female ratios of 37/11 and 35/10, respectively.

In the study by Maurer et al. [18], 345 patients who had surgical site infection-related surgery at a level 1 trauma center were included. They evaluated all patients based on their nutritional status as determined by the Nutritional Risk Screening in 2014/2015 and 2017/2018, and 238 datasets (69.0%) were available for the follow-up analysis. Twenty (8.4%) patients had passed away, totaling 218. At Exam 1, 32.8% of the population was at risk for malnutrition. ROM was greater in female and male patients (p < 0.05). Overall, male dominance was observed in their study.

In the present study, while analyzing the nutritional status of patients per SGA group comparison at admission, we observed that the mean score at admission was higher in Group A (6.25 ± 0.43) than in Group B + C (2.93 ± 1.06) along with a statistically significant difference among them (p < 0.0001). In Group A, most patients were well-nourished (36 (75%)); however, in Group B + C, most patients showed mild-to-moderate malnourishment (21 (46.67%)), with a statistically significant difference among them (p < 0.0001).

The mean score at discharge in our patients was higher in Group A (6.25 ± 0.43) than in Group B + C (2.93 ± 1.06), with a statistically significant difference among them (p < 0.0001). In Group A, most patients were well-nourished (36 (75%)); however, in Group B + C, most patients showed mild-to-moderate malnourishment (21 (46.67%)), with a statistically significant difference among them (p < 0.0001*).

According to a study by Meyer et al. [19], discharge location was unrelated to nutritional status (p = 0.641). Overall, 20 (8.4%) of the 238 available patients died during the follow-up period, resulting in a 6.2-fold increased mortality risk in patients with ROM. The EQ-5D revealed that mobility, self-sufficiency, and typical activities of daily living were enhanced in well-nourished patients (p < 0.001).

In this study, we observed that the mean score in six months was higher in Group A (6.65 ± 0.48) than in Group B + C (4.93 ± 1.34), with a statistically significant difference among them (p < 0.0001). In Group A, most patients were well-nourished (31 (64.58%)); similarly, in Group B + C, most patients were well-nourished (24 (53.33%)), with a statistically significant difference among them (p < 0.0001).

Maximum complications were observed in Group B + C than in Group A. Further, in Group A, the maximum number of patients reported having decubitus (38 (79.17%)), followed by wound infection (16 (33.33%)), and so on. Whereas in Group B + C, the maximum number of patients reported having wound infection (45 (100%)), followed by infected implants, ileus, and others (18 (40%)). Furthermore, a statistically significant difference was observed in both groups for all observed complications.

The mean RUST score for six months in Group A (9.94 ± 0.66) was more than the score in Group B + C (8.67 ± 0.79), with a statistically significant difference among them (p < 0.0001). Also, most patients reported for SGA-A at six months (72), for SGA-B at admission, discharge, one week, and one month (36 each). Lastly, for SGA-C at admission, discharge, one week, and one month (nine each).

Additionally, in this study, while analyzing the RUST score with various parameters at six months, we observed a positive correlation with a significant association between the RUST score versus serum bioalbumin, hemoglobin, platelets count, TLC, at admission nutritional status, at discharge nutritional status, at one-week nutritional status, at one-month nutritional status, at three-month nutritional status, but a negative correlation with a significant association was observed between the RUST score versus CRP.

Based on the findings of this study, we have reported that a significant percentage of people undergoing orthopedic surgery were either malnourished or at risk of becoming malnourished. Postoperative problems in patients undergoing orthopedic surgery are more common when hunger or the possibility of malnutrition exists. However, to bypass the confounder, we recommend a well-designed, resilient, multicentric study with a higher sample size.

Limitations

This study had a few limitations. The sample size of 93 was comparatively moderate. A larger sample size and multicentric study design with high precision and accuracy are recommended for a more reliable interpretation of results. One of the major limitations of our study was the nutritional assessment, as SGA has some limitations as a reference assessment tool. In this study, the nutrition assessment was performed by a single examiner to exclude interobserver differences. A limitation of SGA is that it only categorizes subjects into three broad groups and does not reveal subtle variations in nutritional status. Results were limited to a single tertiary care center that may not be generalized for all settings. Hence, it cannot be incorporated into a larger population. Further longitudinal studies should be conducted assessing similar concerns of malnutrition status and its effect on outcomes in patients with limb injuries using the SGA as a screening tool.

## Conclusions

Malnutrition is a major problem among hospitalized patients and is associated with increased rates of morbidity and mortality, cost, and duration of hospital stay. Thus, hospitalized individuals should have their nutritional status examined. In the present study, we assessed the assessment of nutrition status and its effect on outcomes in patients with limb injuries by SGA as a screening tool. Based on the findings of this study, we may conclude that malnutrition significantly increases the chance of developing complications such as wound infection, decubitus, and infected implants.

In this study, we observed a statistical difference in the serum biomarker levels of albumin, total lymphocyte count, CRP, and platelet count in well-nourished and malnourished patients, which has a significant association independently with infectious and non-infectious complications. We observed that clinical and radiological bone healing, which was measured by the RUST scoring system, had a positive correlation with the nutritional status of the patients.
